# Dental Microwear and Diet of the Plio-Pleistocene Hominin *Paranthropus boisei*

**DOI:** 10.1371/journal.pone.0002044

**Published:** 2008-04-30

**Authors:** Peter S. Ungar, Frederick E. Grine, Mark F. Teaford

**Affiliations:** 1 Department of Anthropology, University of Arkansas, Fayetteville, Arkansas, United States of America; 2 Department of Anthropology, Stony Brook University, Stony Brook, New York, United States of America; 3 Department of Anatomical Sciences, Stony Brook University, Stony Brook, New York, United States of America; 4 Leverhulme Center for Human Evolutionary Studies, The University of Cambridge, Cambridge, United Kingdom; 5 Center for Functional Anatomy and Evolution, The Johns Hopkins University School of Medicine, Baltimore, Maryland, United States of America; University of Cambridge, United Kingdom

## Abstract

The Plio-Pleistocene hominin *Paranthropus boisei* had enormous, flat, thickly enameled cheek teeth, a robust cranium and mandible, and inferred massive, powerful chewing muscles. This specialized morphology, which earned *P. boisei* the nickname “Nutcracker Man”, suggests that this hominin could have consumed very mechanically challenging foods. It has been recently argued, however, that specialized hominin morphology may indicate adaptations for the consumption of occasional fallback foods rather than preferred resources. Dental microwear offers a potential means by which to test this hypothesis in that it reflects actual use rather than genetic adaptation. High microwear surface texture complexity and anisotropy in extant primates can be associated with the consumption of exceptionally hard and tough foods respectively. Here we present the first quantitative analysis of dental microwear for *P. boisei*. Seven specimens examined preserved unobscured antemortem molar microwear. These all show relatively low complexity and anisotropy values. This suggests that none of the individuals consumed especially hard or tough foods in the days before they died. The apparent discrepancy between microwear and functional anatomy is consistent with the idea that *P. boisei* presents a hominin example of Liem's Paradox, wherein a highly derived morphology need not reflect a specialized diet.

## Introduction

*Paranthropus boisei* has the biggest, flattest cheek teeth, and the thickest dental enamel of any known member of our tribe, the Hominini [1], [2]. It's cranium and mandible appear built to resist the stresses associated with heavy chewing, and provide copious attachment areas for massive muscles of mastication [3]–[6]. It is no surprise then that *P. boisei* has been widely considered to have been a hard-object feeder, specializing on nuts and seeds, or on roots and tubers from the savannas that spread throughout eastern Africa during the Plio-Pleistocene [7]. That said, craniodental functional morphology offers insights into what a hominin *could* have eaten, but not necessarily what it actually ate on a regular basis. By contrast, dental microwear, the pattern of microscopic use-wear on a tooth, is caused by, and reflects, specific foods eaten by the individual whose teeth are being examined. Thus, microwear can provide direct evidence for the diets and foraging strategies of fossil species.

Patterns of dental microwear reflect the physical properties of foods eaten. Thus, primates that consume hard, brittle foods tend to have heavily pitted, complex microwear surface textures, whereas those that eat tough leaves or stems have more anisotropic surfaces dominated by long, parallel striations [8], [9]. Microwear can be assessed accurately by combining scanning confocal profilometry and scale-sensitive fractal analysis to characterize microscopic surface texture attributes, such as complexity and anisotropy, in three dimensions [9]–[11]. This approach, called microwear texture analysis, eliminates observer error inherent in feature-based measurements, thereby allowing more confident comparisons of *distributions* of data in addition to standard statistical analyses of central tendencies. Given that microwear forms and changes quickly (i.e., in the days before death) [12], it becomes possible to consider the ranges of foods eaten by a species, rather than just the most commonly-eaten foods implied by such labels as “folivore” or “frugivore”.

While no study to date has focused on dental microwear textures of *Paranthropus boisei*, its South African congener, *Paranthropus robustus* has been examined. Although early microwear study hinted at a diet dominated by hard, brittle objects [13], more recent texture analysis suggests that *P. robustus* ate such foods only periodically throughout the year [11]. Such dietary flexibility is consistent with recent isotope analyses [14], measures of occlusal surface topography [15], behavioral-ecological models based on living African apes [16], [17] and paleoecological data on the food resources available at the time [18]. The idea is that *P. robustus* “fell back” on less preferred, mechanically challenging items at times of resource stress when preferred foods were unavailable, much like modern-day lowland gorillas do with tough foods. The notion that morphological specializations seen in *Paranthropus* act to increase diet breadth by allowing the consumption of hard, brittle foods without compromising the ability to consume softer, weaker ones is consistent with Liem's Paradox. This dictum, originally developed from studies of fish, states that specialized morphology can allow for a broader diet wherein a species may actively avoid the very foods to which it is adapted when other, more preferred resources are available [19], [20].

This begs the question “what about the most craniodentally specialized of the early hominins, *Paranthropus boisei*?” Conventional wisdom suggests that the adaptive morphology of *P. boisei* was so derived that it must have been a dietary specialist [7], [21] (Fig S1). Its large, flat occlusal surfaces combined with thick enamel and massive, anteriorly positioned jaw elevators has led most investigators to infer a diet dominated by hard, brittle foods, such as seeds or underground storage organs [1], [22] (Fig S2). It is no wonder then that the nickname “Nutcracker Man” is still used for this hominin nearly half a century after it was introduced.

Others have noted that powerful muscles combined with large chewing platforms may have, in essence, balanced out, resulting in masticatory stresses similar to those of other hominins, albeit distributed over a larger occlusal surface [23], [24]. This has suggested to some that hominin “megadontia” reflects repetitive loading of large quantities of lower energy, tougher foods. This model sits in contrast to the observation that large cheek teeth are well-suited, biomechanically, for chewing small or thin foods [25], [26]. In the end, as Constantino and Wood [27] recently lamented, “there has not been much success in determining the diet of *P. boisei*”.

Dental microwear is well-suited to evaluating such models as it offers direct evidence of the mechanical properties of food items eaten by individuals during life. While early workers suggested the potential of microwear for the inference of *Paranthropus boisei* diets [23], [28] there has been no comprehensive, quantitative study of this taxon. This is surprising because microwear patterns are especially valuable for distinguishing extant hard-object feeders from tough food eaters. Such data could also permit comparison of *P. boisei* with its congener, *P. robustus*. It has been suggested that the two species may have been ecological vicars, playing similar ecological roles during the Plio-Pleistocene in eastern Africa and South Africa respectively [29].

Here we evaluate two hypotheses using dental microwear texture analysis: 1) *Paranthropus boisei* regularly consumed mechanically challenging foods (hard or tough); and 2) *Paranthropus boisei* and *P. robustus* had similar diets. This analysis focused on Facet #9 of all permanent molars of *P. boisei* available to us. This facet is located on the crushing/grinding (“Phase II”) surface, an area that has shown consistent and predictable differences in microwear patterns between extant primates with differing diets [8], [9]. Only seven of fifty-three numbered individuals examined preserved unobscured antemortem microwear (KNM-CH 1 from Chesowanja, Kenya, KNM-ER 729, 3230 and 3952 from Koobi Fora, Kenya, KNM-WT 17400 from West Turkana, Kenya, OH 5 from Olduvai Gorge, Tanzania, and L7A-125 from the Omo, Ethiopia). Nevertheless, these seven fossils span most of the known geochronological range of *P. boisei*, from as early as 2.27 Myr to as recently as about 1.4 Myr. The environments in which they lived are reconstructed as having been dominated by grasslands, but also some more closed, wet habitats associated with riverine and lacustrine elements (see supporting information Text S1 and Table S1).

Data on surface fractal complexity (*Asfc*) and anisotropy (*epLsar*), two texture attributes known to distinguish living primates with different diets, were collected and compared with those previously published for extant primates [9] and early hominins [11]. The extant baseline taxa represent two species known to consume, at least on occasion, hard objects (*Cebus apella* and *Lophocebus albigena*) and two that eat tougher foods including leaves and stems (*Alouatta palliata* and *Trachypithecus cristata*). The other fossil hominins used for comparison with *P. boisei* include *Australopithecus africanus* and *Paranthropus robustus*, both from the Plio-Pleistocene of South Africa.

## Results

**Figure 1:**
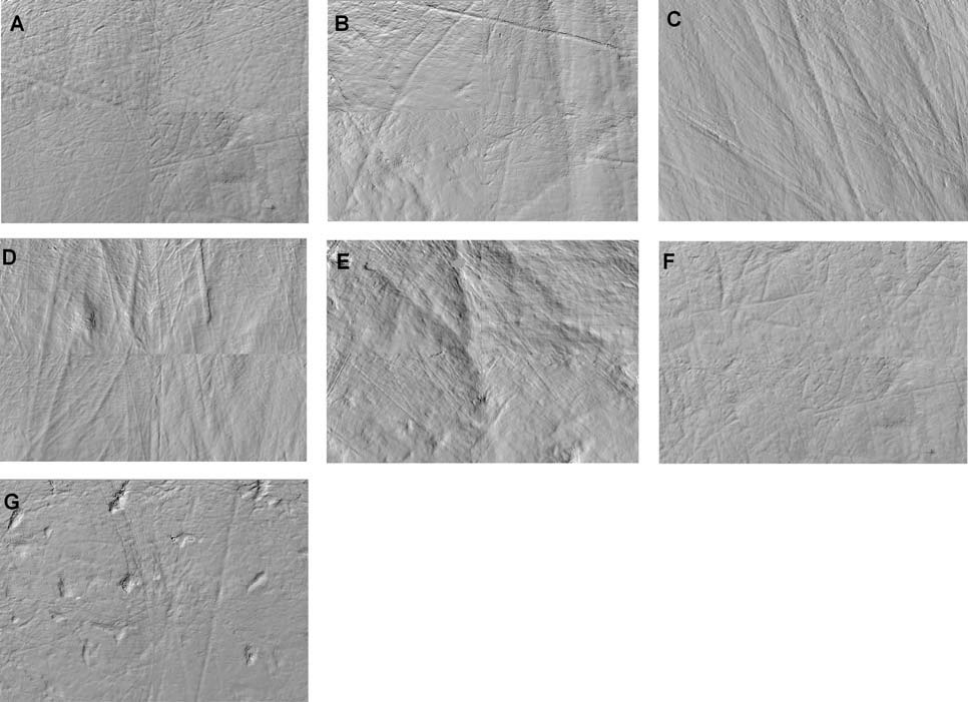
Photosimulation montages of all *Paranthropus boisei* specimens known to preserve antemortem microwear. Each montage is comprised of photosimulations of 3D point clouds for four adjacent fields representing a total of 276×204 µm of each original occlusal surface. (A) KNM-CH 1, (B) KNM-ER 729, (C) KNM-ER 3230, (D) KNM-ER 3952, (E) KNM-WT 17400, (F) OH 5, (G) Omo L7A-125.

All *Paranthropus boisei* specimens had light microwear, with most showing wear surfaces dominated by fine striations (Fig 1). None had the large, deep pits expected of a hard-object specialist or the uniformly large, deep and parallel striations observed for tough food grazing mammals. Fractal complexity values were uniformly low with minimal variation, and anisotropy values were moderate, both in range and central tendency.

**Figure 2:**
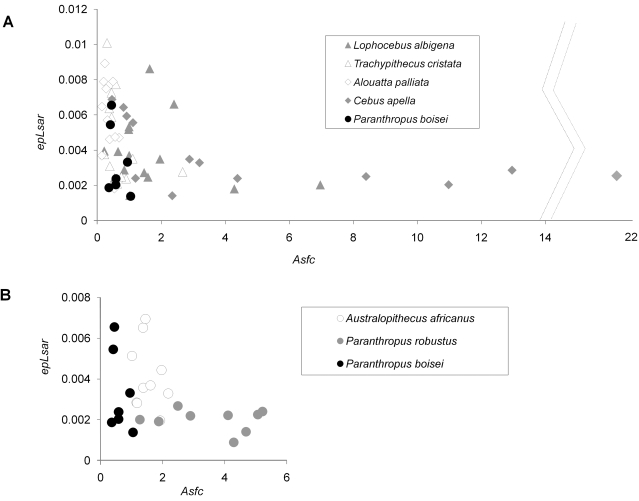
Dental microwear comparisons of *Paranthropus boisei* individuals with (A) South African early hominins and (B) various extant species. The x-axis and y-axis represent surface complexity (*Asfc*) and anisotropy (*epLsar*) respectively.

*Paranthropus boisei* fractal complexity values fell near the bottom end of the range for living primates. None showed the extremely high *Asfc* values observed for some *Lophocebus albigena* and especially *Cebus apella* individuals. Further, none of the *P. boisei* individuals showed the extremely high anisotropy values reported for some *Trachypithecus cristata* and *Alouatta palliata* individuals (Fig 2a). These results are borne out to a degree by statistical analyses despite the small sample size for the fossil hominin (Tables 1–2). Specifically, *P. boisei* had significantly lower *Asfc* values and variance than *C. apella*, and marginally lower *Asfc* values than *L. albigena*. Marginally lower is here defined as *p*≤0.05 for Fisher's LSD but not Tukey's HSD tests. The hominin also had marginally higher *Asfc* values and lower *epLsar* values than *A. palliata*.

**Table 1:** Comparisons of *Paranthropus boisei* with extant species.

	*F*	*df*	*p*
Central tendencies			
MANOVA Wilks' λ	7.18	8, 104	0.00
ANOVA *Asfc*	14.98	2, 23	0.00
ANOVA *epLsar*	2.50	2, 23	0.05
Sample variances (Levene's Test)			
MANOVA Wilks' λ	6.20	8, 104	0.00
ANOVA *Asfc*	13.85	4, 53	0.00
ANOVA *epLsar*	0.95	4, 53	0.44

**Table 2:** Pairwise comparisons of *Paranthropus boisei* and extant species.

	*L. albigena*	*C. apella*	*P. boisei*	*T. cristata*
*Asfc* central tendencies				
*C. apella*	8.06			
*P. boisei*	−14.11†	−22.18‡		
*T. cristata*	−15.15‡	−23.21‡	−1.04	
*A. palliata*	−25.67‡	−33.73‡	−11.558†	−10.523†
*epLsar* central tendencies				
*C. apella*	−1.45			
*P. boisei*	−6.60	−5.15		
*T. cristata*	6.32	7.76	12.92	
*A. palliata*	14.31†	15.76†	20.91†	7.99
*Asfc* Sample variances (Levene's Test)				
*C. apella*	3.77‡			
*P. boisei*	−0.93	−4.70‡		
*T. cristata*	−0.72	−4.49‡	0.20	
*A. palliata*	−0.98	−4.75‡	−0.06	−0.26

‡ Tukey's HSD test *p*≤0.05

† Fisher's LSD test *p*≤0.05

The comparisons with other early hominins are even more telling (Fig 2b). The points on a bivariate plot of *Asfc* and *epLsar* for *Paranthropus boisei* clustered separately from both *Australopithecus africanus* and *P. robustus.* While the distribution of *epLsar* values for *P. boisei* closely matched that for *A. africanus*, the two showed no overlap in *Asfc*, with the former having lower values than the latter in all cases. The differences in both *Asfc* and *epLsar* between *P. boisei* and *P. robustus* were also remarkable, with the eastern African “robust” form having much lower *Asfc* values and ranges and higher average *epLsar* values and ranges than its South African counterpart. These differences were borne out in statistical analyses, despite small sample sizes (Tables 3–4). *Paranthropus boisei* had significantly lower *Asfc* values than both *A. africanus* and *P. robustus*, significantly lower *Asfc* variance than *P. robustus*, and significantly higher *epLsar* variance than *P. robustus.*

**Table 3:** Comparisons of fossil species.

	*F*	*df*	*p*
Central tendencies			
MANOVA Wilks' λ	8.9	4, 44	0.00
ANOVA *Asfc*	23.18	2, 23	0.00
ANOVA *epLsar*	5.25	2, 23	0.01
Sample variances (Levene's Test)			
MANOVA Wilks' λ	8.49	4, 44	0.00
ANOVA *Asfc*	20.21	2, 23	0.00
ANOVA *epLsar*	6.18	2, 23	0.01

**Table 4:** Pairwise comparisons of fossil species.

	*Asfc*		*epLsar*	
	*A. africanus*	*P. boisei*	*A. africanus*	*P. boisei*
Central tendencies				
*P. boisei*	−9.26‡		−5.46	
*P. robustus*	27.49‡	16.75‡	−10.49‡	−5.03
Sample variances (Levene's Test)				
*P. boisei*	−3.07		1.23	
*P. robustus*	11.06‡	14.13‡	−8.76 ‡	−9.98‡

‡ Tukeys HSD test *p<0.05*

Comparisons with the extant baseline series suggest that none of the *Paranthropus boisei* individuals examined consumed extremely hard or extremely tough foods in the days before death. All of these specimens lacked the extremes of *Asfc* evinced by *Lophocebus albigena* and especially *Cebus apella*, both known to consume hard, brittle foods. *Paranthropus boisei* molars also lacked the extremes of *epLsar* seen in *Trachypithecus cristata* and *Alouatta palliata*, both known to consume tough leaves and stems. The *P. boisei* individuals examined evidently avoided such metabolically challenging foods, at least in the days before death. This is notably consistent with Walker's [23] early assertion that *P. boisei* microwear patterns resemble those of living frugivores, and differ from those of living grazers, leaf browsers, and bone feeders.

Comparisons with the South African hominins suggest that while *Paranthropus boisei* may have consumed foods with similar ranges of toughness as those eaten by *Australopithecus africanus*, the eastern African “robust” hominin did not eat harder and brittler foods than the South African “gracile” form. Further, the patterns for *P. boisei* and *P. robustus* are very different. *Paranthropus robustus* likely ate foods that were on average much harder and less tough than *P. boisei*. The differences in both central tendencies and ranges of variation suggest different feeding strategies, and by implication, that the two species of *Paranthropus* probably had markedly different diets or foraging strategies.

## Discussion

While the craniodental functional morphology *Paranthropus boisei* suggests an ability to generate and dissipate forces associated with the consumption of extremely hard or tough foods, microwear texture analysis offers no evidence that these hominins regularly did so. Thus, there is an apparent discordance between microwear and biomechanical models based on craniodental functional morphology. The resolution of this discordance probably lies in fundamental differences in the nature of genetic and non-genetic signals for diet. While adaptive morphology gives important clues as to what an animal is (or was) was capable of eating, microwear reflects what an animal actually did eat at some point during its lifetime. While the craniodental features of *Paranthropus boisei* would have been capable of generating large forces on small objects, or processing large quantities of tough, fibrous foods, microwear suggests that the individuals examined did not do so in the days before death.

There are several possible explanations for this discrepancy. It may be that the combination of microwear and morphology point to a novel type of diet difficult to identify given a lack of extant analogs among the primates. While this is possible, such lines of reasoning are unhelpful because, without the comparative method, paleobiological interpretation may be left in “undecipherable chaos” [30]. Another possibility is that the specimens examined are not representative of the species as a whole. While the vast majority of specimens in the hypodigm were excluded from this study because of taphonomic damage, the uniformity of texture patterns for all seven useable specimens is remarkable, especially given their separation in time and geography. It is difficult to imagine that none of these specimens would show complex or highly anisotropic microwear surfaces if the species regularly consumed extremely hard or tough foods.

A final possibility is that *Paranthropus boisei* did occasionally consume extremely hard or tough foods, but did so sufficiently rarely that it was not picked up in the microwear of the seven individuals sampled. This would suggest a model akin to the subsistence pattern of gorillas that prefer nutrient rich soft fruits but fall back on less desirable, more difficult to digest stems and leaves at “crunch times” [16], [17], [22]. If so, *P. bosei* would present another example of Liem's Paradox. Robinson and Wilson [19] wrote that “some resources are intrinsically easy to use and are widely preferred, while others require specialized phenotypic traits on the part of the consumer. This asymmetry allows optimally foraging consumers to evolve phenotypic specializations on non-preferred resources without greatly compromising their ability to use preferred resources… Specialists should often reject the very resources that they have evolved traits to use” (p. 223).

The differences between *Paranthropus boisei* and *P. robustus* microwear patterns are more difficult to interpret in this light. *Paranthropus robustus* has a microwear pattern similar to those of *Lophocebus albigena* and *Cebus apella*, two living primates that fall back on hard, brittle foods when less mechanically challenging, preferred resources are unavailable. A hard-object fallback model for *P. robustus* gains considerable support from recent studies of isotopes, occlusal morphology, living African apes, and plant resources available in the Plio-Pleistocene [14]–[18]. If *Paranthropus boisei* craniodental morphology also reflects fallback exploitation, they likely consumed extremely hard or tough foods even less frequently than did their South African congeners.

## Materials and Methods

We first made dental impressions of molar teeth of *Paranthropus boisei* available in the National Museums of Kenya and Ethiopia in Nairobi and Addis Ababa, respectively in the 1990s. These included, with the addition of OH 5 from the National Museums of Tanzania in Dar-es-Salaam (courtesy of Alejandro Pérez-Pérez), all erupted permanent molars preserving crown enamel for the entire hypodigm of *Paranthropus boisei* at the time (see Table S2). Original specimens were cleaned with cotton swabs soaked in alcohol and crown surface molds were prepared using President's Jet regular body polyvinylsiloxane dental impression material (Coltène-Whaledent Corp.). Tooth replicas were then poured using Epotek 301 epoxy resin and hardener (Epoxy Technologies Corp.).

Replicas were then examined by light microscopy to determine suitability for microwear analysis. Thirty-three candidate specimens were then examined at higher resolution using a Sensofar PLµ confocal imaging profiler (Solarius, Inc.). Unfortunately, based on standard assessments of postmortem wear [31], [32] only seven molars preserved unambiguous, unobscured antemortem microwear on their “Phase II” facets and could be included in this analysis. These include KNM-CH 1 from Chesowanja, Kenya, KNM-ER 729, 3230 and 3952 from Koobi Fora, Kenya, KNM-WT 17400 from West Turkana, Kenya, OH 5 from Olduvai Gorge, Tanzania, and L7A-125 from the Omo, Ethiopia.

Three-dimensional point clouds were generated using confocal profilometry for Facet #9 of each specimen at a lateral (x, y) interval of 0.18 µm with a vertical resolution of 0.005 µm. Four adjacent fields of 138 µm×102 µm were sampled for a total area of 276 µm×204 µm. Each point cloud was analyzed using scale-sensitive fractal analysis software (ToothFrax and SFrax, Surfract Corp). We focused this study on fractal complexity (*Asfc*) and anisotropy (*epLsar*) as these measures had previously proven useful in distinguishing among primates with different diets [9], [33]. Complexity is measured as change in surface roughness at different scales, so a surface dominated by pits of various sizes or pits and scratches will tend toward relatively high complexity. Anisotropy is a measure of orientation concentration of surface roughness, so a facet dominated by striations running parallel to one another will have high anisotropy. Median values of *Asfc* and *epLsar* for the four fields representing each specimen were computed and used in subsequent analyses.

Two sets of statistical analyses were conducted, one to compare *Paranthropus boisei* with extant primates with known differences in diet, and the other to compare *Paranthropus boisei* with other early hominins. The extant baseline data were originally published and described by Scott et al.[9]. These include two “tough food” eaters, *Alouatta palliata* (*n* = 11) and *Trachypithecus cristata* (*n* = 12) and two “hard-object” fallback feeders, *Cebus apella* (*n* = 13) and *Lophocebus albigena* (*n* = 15). These were chosen as contrasting pairs of New World and Old World monkeys, each exhibiting more emphasis on hard or tough foods than is found in modern chimpanzees or gorillas. The comparative sample of fossils was originally presented and described by Scott et al [11], and includes South African *Australopithecus africanus* (*n* = 10) and *Paranthropus robustus* (*n = *9) from Sterkfontein and Swartkrans respectively.

First, multivariate analyses of variance (MANOVAs) on ranked [34] *Asfc* and *epLsar* data was were used to compare taxa for both sets of analyses. Sources of significant variation were then assessed by individual ANOVAs on each variable, and multiple comparisons tests as necessary. Both Fisher's LSD *and* Tukey's HSD tests were used to balance risks of Type I and Type II errors [35].

Raw data for each variable were then transformed for Levene's Test following the procedure described by Plavcan and Cope [36] to compare distribution variances between taxa. The same procedure used for comparisons of the ranked data, MANOVAs followed by ANOVAs and multiple comparisons tests, was used to assess significance of differences between taxa in variance of *Asfc* and *epLsar* values.

## Supporting Information

Text S1 This document describes the paleoenvironmental contexts of the specimens analyzed in this study
(0.06 MB DOC)

Table S1 Geochronological age of *Paranthropus boisei* specimens employed in this study.
(0.03 MB DOC)

Table S2 Specimens of *Paranthropus boisei* examined for this study.
(0.04 MB DOC)

Figure S1 Cranium of *Paranthropus boisei* (OH 5). Image courtesy of Donald C. Johanson.
(4.76 MB TIF)

Figure S2 Palate and maxillary teeth of *Paranthropus boisei* (OH 5). Image courtesy of Donald C. Johanson.
(1.54 MB TIF)
