# Correction: Dental Microwear and Diet of the Plio-Pleistocene Hominin *Paranthropus boisei*

**DOI:** 10.1371/annotation/195120f0-18ee-4730-9bd6-0d6effd68fcf

**Published:** 2008-05-02

**Authors:** Peter S. Ungar, Frederick E. Grine, Mark F. Teaford

There was an error in the author affiliations. The correct affiliations should appear as shown here:

Peter S. Ungar^1^, Frederick E. Grine^2,3,4^, Mark F. Teaford^5^

1 Department of Anthropology, University of Arkansas, Fayetteville, Arkansas, United States of America, 2 Department of Anthropology, Stony Brook University, Stony Brook, New York, United States of America, 3 Department of Anatomical Sciences, Stony Brook University, Stony Brook, New York, United States of America, 4 Leverhulme Center for Human Evolutionary Studies, The University of Cambridge, Cambridge, United Kingdom, 5 Center for Functional Anatomy and Evolution, The Johns Hopkins University School of Medicine, Baltimore, Maryland, United States of America

