# Supplementary material for: Dental Microwear and Diet of the Plio-Pleistocene Hominin Paranthropus boisei
Source: PLoS One. 2008 Apr 30;3(4):e2044. doi: 10.1371/journal.pone.0002044 (PMC2315797; doi:10.1371/journal.pone.0002044)
Supplement: Text S1 — This document describes the paleoenvironmental contexts of the specimens analyzed in this study (0.06 MB DOC) [file pone.0002044.s001.doc]

**Supporting Information**

Fossils attributable *Paranthropus boisei* are known from Bed I to close to the top of Bed II of Olduvai Gorge, Tanzania, the Humba Formation at Peninj (Natron), Tanzania, the Chemoigut Formation at Chesowanja, Kenya, the Upper Burgi, KBS, Okote and lower Chari Members of the Koobi Fora Formation, Kenya, the Kaitio Member of the Nachukui Formation, Kenya, Members G through K (and possibly L) of the Shungura Formation, Ethiopia, and at Konso, Ethiopia [1–3]. Thus, this species is recognized to extend from about 2.3 Myr to approximately 1.4 to 1.2 Myr.

The seven specimens attributable to *P. boisei* for which we were able to observe microwear (Table 4) span most of the known geochronological range of that species, with Omo 7A-125 dating to between 2.27 - 2.19 Myr and the Chesowanja hemi-cranium (KNM-CH 1) dating to greater than some 1.4 Myr.

The paleoenvironmental reconstructions that have been proffered for localities and sites from which *Paranthropus boisei* fossils are known range from extensive, dry grasslands to wet, closed habitats with woodlands and perhaps forests. Thus, Shipman and Harris [4] concluded from an analysis of the large-mammal faunas at Olduvai Gorge, Koobi Fora, West Turkana, and the Shungura Formation that *P. boisei* “probably preferred closed over open habitats and favored wetter rather than drier closed habitats.” Reed’s [5] study of the ecomorphology of associated bovids led her to conclude that at Koobi Fora, *P. boisei* was found in more open habitats, and with the presence of edaphic grasslands.

In particular, studies of pollen, micromammals and large mammal remains from Member G of the Shungura Formation suggest varied pictures, ranging from closed, wet habitats [4] to open woodlands and edaphic grasslands [5,6], to arid Acacia grasslands. The Konso *P. boisei* fossils are associated with a predominantly dry grassland fauna, and are notably absent from more mesic localities that have been sampled in the proximate area [3,7].

Beds I and II of Olduvai Gorge has been characterized as sampling a lake margin woodland and forest to open grasslands in the lower and higher stratigraphic profiles [8,9]. Shipman & Harris [4] suggested that the fauna sampled open, arid as well as closed, wet habitats, while Blumenshine et al. [10] observed the presence of grassland plants with gallery forest in vicinity of streams.

Bishop et al. [11] suggested that the fauna from the Chemoigut Formation at Chesowanja characterized a bushed - grassland habitat, with riverine as well as lacustrine elements. Paleoenvironmental reconstructions have not been provided for Peninj. The palynological analysis of Dominguez-Rodrigo et al. [12] at Peninj was confined to the Upper Sands, whereas the *P. boisei* mandible derives from the older Basal Sands.

Reference List

1. Suwa G (1988) Evolution of the "robust" australopithecines in the Omo succession: Evidence from mandibular premolar morphology. In: Grine FE, editors. Evolutionary History of the 'Robust' australopithecines. New York: Aldine de Gruyter. pp.

2. Suwa G, White TD, Howell FC (1996) Mandibular postcanine dentition from the Shungura Formation, Ethiopia: Crown morphology, taxonomic allocations, and Plio-Pleistocene hominid evolution. Am J Phys Anthrop 101: 247-282.

3. Suwa G, Asfaw B, Beyene Y, White TD, Katoh S, et al. (1997) The first skull of *Australopithecus boisei*. Nature 389: 489-492.

4. Shipman P, Harris JM (1988) Habitat preference and paleoecology of A*ustralopithecus boisei* in eastern Africa. In: Grine FE, editors. Evolutionary History of the 'Robust' australopithecines. New York: Aldine de Gruyter. pp. 343-381.

5. Reed KE (1997) Early hominid evolution and ecological change through the African Plio-Pleistocene. J Hum Evol 32: 289-322.

6. Alemseged Z (2003) An integrated approach to taphonomy and faunal change in the Shungura Formation (Ethiopia) and its implication for hominid evolution. J Hum Evol 44: 451-478.

7. Nagaoka S, Katoh S, WoldeGabriel G, Sato G, Nakaya H, et al. (2005) Lithostratigraphy and sedimentary environments of the hominid-bearing Pliocene-Pleistocene Konso Formation in the southern Main Ethiopian Rift, Ethiopia. Palaeo 216: 333-357.

8. Hay RL (1976) Geology of the Olduvai Gorge. A study of Sedimentation in a Semiarid Basin. Berkeley: University of California Press.

9. Kappelman J (1984) Plio-Pleistocene environments of Bed I and Lower Bed II, Olduvai Gorge, Tanzania. Palaeogeogr Palaeoclimatol Palaeoecol 48: 171-196.

10. Blumenschine RJ, Peters CR, Masao FT, Clarke RJ, Deino AL, et al. (2003) Late Pliocene *Homo* and hominid land use from western Olduvai Gorge, Tanzania. Science 299: 1217-1221.

11. Bishop WW, Hill A, Pickford M (1978) Chesowanja: A revised geological interpretation. In: Bishop WW, editors. Geological Background to Fossil Man. London: Scottish Academic Press. pp. 309-327.

12. Dominguez-Rodrigo M, Lopez-Saes JA, Vincens A, Alcala L, Luque L, et al. (2001) Fossil pollen from the Upper Humbu Formation of Peninj (Tanzania): Hominid adaptation to a dry open Plio-Pleistocene savanna environment. J Hum Evol 40: 151-157.
