# Supplementary material for: Dental Microwear and Diet of the Plio-Pleistocene Hominin Paranthropus boisei
Source: PLoS One. 2008 Apr 30;3(4):e2044. doi: 10.1371/journal.pone.0002044 (PMC2315797; doi:10.1371/journal.pone.0002044)
Supplement: Table S1 — Geochronological age of Paranthropus boisei specimens employed in this study. (0.03 MB DOC) [file pone.0002044.s002.doc]

Table S1. Geochronological age of *Paranthropus boisei* specimens employed in this study.

| Specimen | Approximate Age |
| --- | --- |
| KNM-CH 1 | > 1.42 Myr |
| KNM-ER 729 | 1.55 - 1.49 Myr |
| KNM-ER 3230 | 1.60 - 1.56 Myr |
| OH 5 | 1.85 - 1.79 Myr |
| KNM-WT 17400 | 1.87 - 1.67 Myr |
| KNM-ER 3952 | c. 1.87 Myr |
| Omo 7A-125 | 2.27 - 2.19 Myr |
