# Supplementary material for: Dental Microwear and Diet of the Plio-Pleistocene Hominin Paranthropus boisei
Source: PLoS One. 2008 Apr 30;3(4):e2044. doi: 10.1371/journal.pone.0002044 (PMC2315797; doi:10.1371/journal.pone.0002044)
Supplement: Table S2 — Specimens of Paranthropus boisei examined for this study. (0.04 MB DOC) [file pone.0002044.s003.doc]

Table S2. Specimens of *Paranthropus boisei* examined for this study.

| KNM-CH 1 | KNM-ER 3230 | OH 38 |
| --- | --- | --- |
| KNM-CH 302 | KNM-ER 3737 | Omo 33-6172 |
| KNM-ER 404 | KNM-ER 3885 | Omo 47-46 |
| KNM-ER 729 | KNM-ER 3886 | Omo 47-1500 |
| KNM-ER 733 | KNM-ER 3887 | Omo 76-37 |
| KNM-ER 801 | KNM-ER 3890 | Omo 141-2 |
| KNM-ER 802 | KNM-ER 3952 | Omo 323-896 |
| KNM-ER 810 | KNM-ER 5429 | Omo F203-1 |
| KNM-ER 818 | KNM-ER 5679 | Omo L7A-125 |
| KNM-ER 1467 | KNM-ER 5877 | Omo L74A-21 |
| KNM-ER 1469 | KNM-ER 15930 | Omo L398-14 |
| KNM-ER 1479 | KNM-ER 15950 | Omo L398-266 |
| KNM-ER 1509 | KNM-ER 25520 | Omo L398-630 |
| KNM-ER 1804 | KNM-WT 17396 | Omo L427-7 |
| KNM-ER 1816 | KNM-WT 17400 | Omo L628-2 |
| KNM-ER 1819 | NMT-W64-160 | Omo L628-3 |
| KNM-ER 1820 | OH 5 | Omo L628-9 |
| KNM-ER 2607 | OH 30 |  |
