# Supplementary figures and images for: Dental Microwear and Diet of the Plio-Pleistocene Hominin Paranthropus boisei
Source: PLoS One. 2008 Apr 30;3(4):e2044. doi: 10.1371/journal.pone.0002044 (PMC2315797; doi:10.1371/journal.pone.0002044)

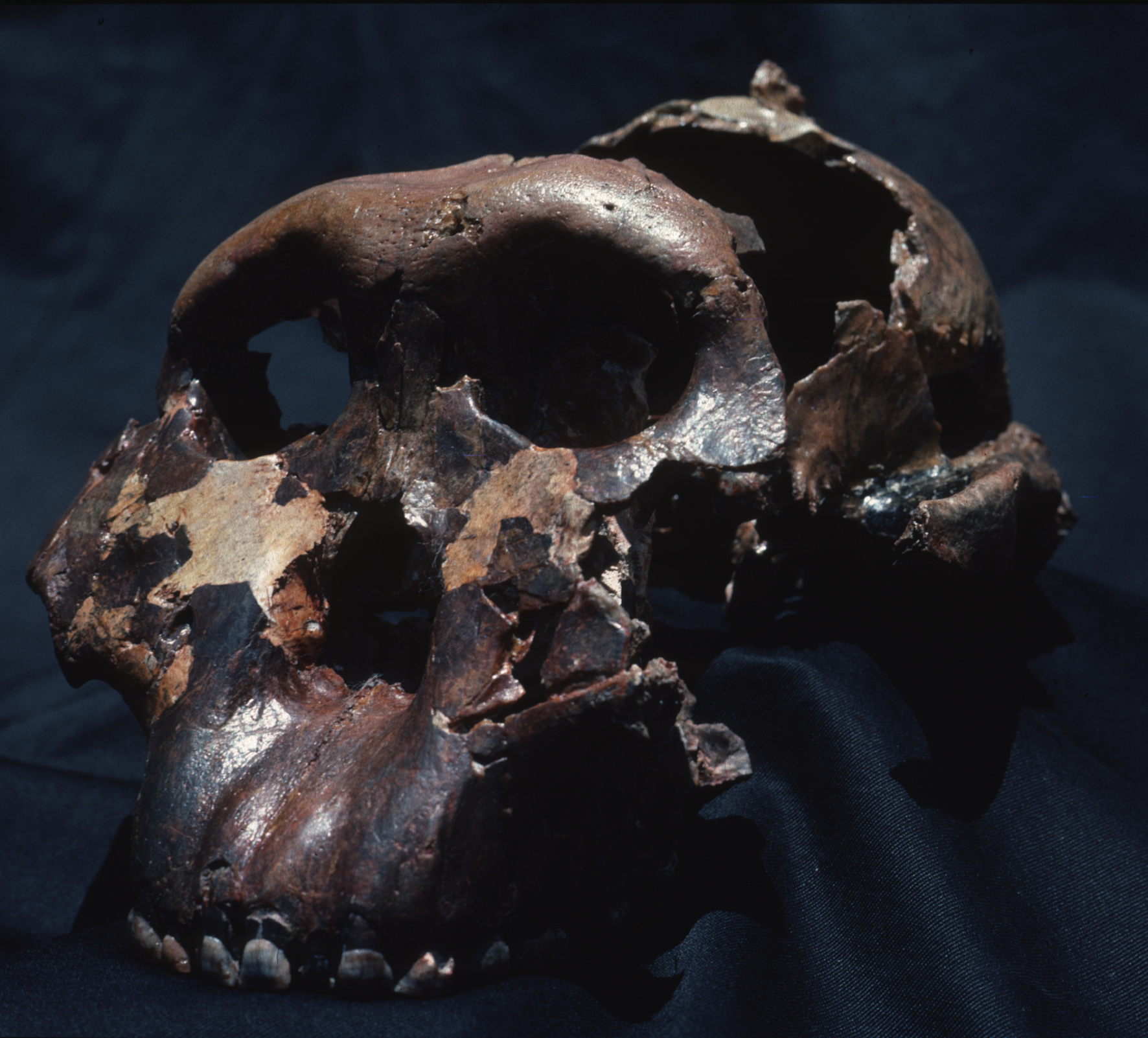

Supplement: Figure S1 — Cranium of Paranthropus boisei (OH 5). Image courtesy of Donald C. Johanson. (4.76 MB TIF) [file pone.0002044.s004.tif]

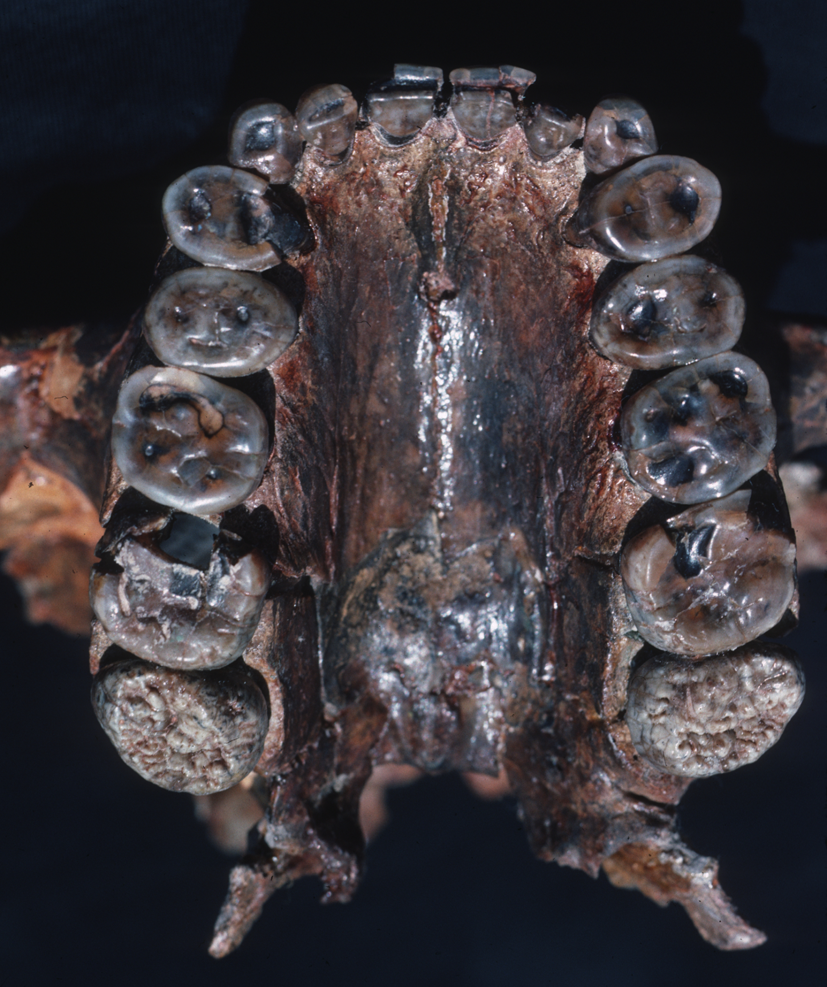

Supplement: Figure S2 — Palate and maxillary teeth of Paranthropus boisei (OH 5). Image courtesy of Donald C. Johanson. (1.54 MB TIF) [file pone.0002044.s005.tif]
